# Object combination in mental simulations

**DOI:** 10.1177/1747021820933214

**Published:** 2020-06-18

**Authors:** Lara N Hoeben Mannaert, Katinka Dijkstra, Rolf A Zwaan

**Affiliations:** Erasmus University Rotterdam, Rotterdam, The Netherlands

**Keywords:** Mental simulation, conceptual combination, grounded cognition, language comprehension

## Abstract

Studies on the presence of mental simulations during language comprehension have typically focused only on single object properties. This study investigates whether two objects are combined in mental simulations, and whether this is influenced by task instructions. In both experiments, participants read sentences describing animals using a tool in some way. After each sentence, they saw an image of a cartoon animal holding a tool, and they indicated whether the animal (Experiment 1) or the tool (Experiment 2) was mentioned in the previous sentence or not. The shown image completely matched, partially matched, partially mismatched, or completely mismatched the preceding sentence. In total, 90 Dutch psychology students took part in Experiment 1, and 92 students took part in Experiment 2, both experiments were pre-registered. The results suggest that mental simulations indeed combine multiple objects during language comprehension and that this is not influenced by task instructions. Regardless of the instruction type, participants always responded quickest in the complete match condition compared to the partial match condition, suggesting that language comprehension leads to the creation of a complete mental simulation.

## Introduction

Mental simulations are defined as the “reenactment of perceptual, motor, and introspective states acquired during experience with the world, body, and mind” (Barsalou, 2008, p. 618). Most studies on mental simulations have used the sentence-picture verification tasks to examine whether single object properties are simulated during language comprehension. For example, when participants read the sentence “The ranger saw an eagle in the sky,” they are faster at responding to a picture of an eagle with spread wings than to an eagle with folded wings (Zwaan et al., 2002). Thus far, researchers have found evidence for the presence of several object properties in mental simulations, including colour (Connell & Lynott, 2009; Hoeben Mannaert et al., 2017; Zwaan & Pecher, 2012), movement (Gentilucci et al., 2000; Glenberg & Kaschak, 2002), orientation (Stanfield & Zwaan, 2001), and size (De Koning et al., 2017). Aside from studies using the sentence-picture paradigm, multiple neuroimaging studies have also found support for modality-specific sensorimotor and affective system activation during language comprehension (Binder & Desai, 2011; Hauk et al., 2004; Sakreida et al., 2013; Simmons et al., 2007).

Interestingly, no studies have yet explored how mental simulations are affected when multiple objects are included in a sentence, as most studies have included items referring to a single object only (e.g., an eagle with spread wings). Researchers tend to assume that for language comprehension to occur, a situation model is built which represents the meaning of the text (Van Dijk & Kintsch, 1983; Zwaan & Radvansky, 1998). However, for a situation model to be complete, it should include a complete representation of the situation described by the text, but this has never been examined before. As past research has only used sentences with one object, we can only know for certain that a mental simulation includes one object. The question that remains then is: when we are reading a text, do we create separate mental simulations for each object that we encounter? Or do we combine multiple objects in a mental simulation to construct a comprehensive situation model? Although we believe this is likely to be the latter, this has never been tested empirically before.

The literature that has come closest to examining this is the literature on conceptual combination, defined as the ability to create new meaning out of already existing concepts (Lynott & Connell, 2010). The Embodied Conceptual Combination (ECCo) model proposed by Lynott and Connell (2010) builds on the idea from the Language and Situated Simulation (LASS) theory (Barsalou et al., 2008) that both linguistic and perceptual information are required for concept representations, and propose the ECCo model as an explanation for how conceptual combination operates. The first principle of the theory states that, similar to the LASS theory, first the linguistic system is activated, which can be used for superficial processing tasks (e.g., when asked to verify the object properties “lemon-yellow,” this would only require the linguistic system to activate due to the strong word associations of those concepts, Solomon & Barsalou, 2004). This is followed by the activation of the simulation system, which is needed for more deep conceptual processing (e.g., when concepts are combined in novel ways, the linguistic system is insufficient to deal with this). This principle was tested in a study by Louwerse and Connell (2011), who examined whether the response times in a property-verification task could be explained by linguistic associations or a simulation approach. This was done by testing whether switching between “linguistic modalities” (i.e., visual-haptic, auditory, and olfactory-gustatory) could predict faster responses than switches between perceptual modalities (i.e., visual, haptic, auditory, olfactory, and gustatory). The linguistic modalities were determined by using word co-occurrences to predict which modality an adjective belonged to. Indeed, they found that the linguistic factors best predicted short response times, while the perceptual factors best predicted longer response times, suggesting that the linguistic system peaks in activation before the perceptual simulation system. It should be noted that these findings were obtained in word-level tasks in which words are presented in isolation. It is not clear yet whether the temporal precedence of linguistic factors can be summarily extrapolated to larger stretches of text in which perceptual representation may have already been activated by prior text once a target word is processed.

The second principle of the ECCo model is that the head and modifier concepts reduce the number of affordances that can be integrated (or “meshed”; cf. Glenberg, 1997) in a simulation. In other words, the number of ways in which an object can interact with another is reduced during this “meshing” (Glenberg, 1997). This meshing can be completed in a destructive or a non-destructive way. If a conceptual combination is destructive, it means that either the modifier or head concept is significantly reduced, such as a *cactus beetle* being interpreted as a green and spiky beetle (Lynott & Connell, 2010). In this case, the *cactus* concept was destroyed and has modified the head concept. In a non-destructive conceptual combination, both concepts remain intact in the simulation. In this case, a *cactus beetle* could be a beetle that feeds on cacti (Lynott & Connell, 2010). The linguistic system interacts with the simulation system by constraining which affordances are plausible (based on past experiences), and also helps to determine early on whether a destructive or non-destructive combination will take place. So when a novel compound noun is encountered, the linguistic information associated with the compound noun is activated first, which then activates the simulation system that can then provide further feedback to the linguistic system, continuing until the process of conceptual combination is complete (Connell & Lynott, 2011).

Several behavioural experiments have provided evidence for the perceptual nature of conceptual combinations. For example, Wu and Barsalou (2009) tested whether participants generated more properties of occluded or unoccluded features in both familiar and novel conceptual combinations. If participants use the simulation system to create conceptual combinations, they would generate more unoccluded properties than occluded properties. Indeed, they found that when participants read, for example, the noun “lawn,” they produced more unoccluded features such as *soft* and *green* compared to occluded features such as *roots* or *dirt*. Conversely, when they read the noun phrase “rolled up lawn,” *roots* and *dirt* became unoccluded, and were thus produced more frequently than *soft* and *green*. As such, it appears as though multiple object properties are simulated when a concept is activated, but that the contents of the simulation may be constrained by the linguistic system.

Connell and Lynott (2011) similarly examined the role of simulations when creating new concepts, where they gave participants a forced-choice interpretation task for novel noun–noun compound phrases (e.g., “octopus apartment”), where they had to answer as quickly as possible whether they could come up with an interpretation for the phrase or not, before providing the interpretation. The results showed that participants respond significantly slower when a destructive interpretation is used compared to a non-destructive interpretation. For instance, if *octopus apartment* is interpreted in a destructive manner, then it could be interpreted as “an apartment with eight rooms,” while a non-destructive interpretation could be “a place where an octopus might live” (Connell & Lynott, 2011, p. 4). It appears then that the integration of multiple objects in a situation model is easier than the manipulation of objects in the situation model, which appears to require more processing time.

As mentioned earlier, conceptual combination refers to the formation of a new concept from existing concepts. As such, most studies on conceptual combination have focused on how compound nouns are processed. In our study, we are interested in seeing whether multiple objects are combined in a mental simulation, but we believe that this combination works through the same mechanism as proposed by the ECCo theory. For example, if a person reads about a bear grabbing a broom from the corner in a room, we believe that the simulation system would activate and simulate a bear holding a broom. This would work similarly to the processes involved in non-destructive conceptual combination, as none of the concepts need to be reduced or “destroyed.” To not confuse terminology, we refer to these types of sentences as involving object combinations, rather than conceptual combinations.

To our knowledge, no studies have yet been published on whether multiple objects are represented in mental simulations, and whether task instructions modulate these simulations. If unfamiliar object combinations are processed in a manner similar to what is proposed by the conceptual combination theories, then we would expect that, in our bear and broom example, both the bear and the broom would be present in the mental simulation. However, what if participants are told to either respond to the animal or to the tool, would they still simulate both objects, or just the one they were told to attend to? A study by Lebois et al. (2015) suggests that the differences in the task instructions would affect the contents of the mental simulation. In their study, participants in one condition were instructed to pay attention to the verticality of words (e.g., “sky” or “basement”) when responding with upward or downward responses based on word colour. In the other condition, participants were uninformed about the manipulation. Their study found only a congruency effect when participants paid attention to verticality, but found no significant effect when participants were unaware of the manipulation. These findings suggest that task instructions can modulate concept activation, and thus it is possible that influencing what comprehenders attend to while reading similarly modulates the contents of their mental simulations.

If a sentence is processed only superficially, and one only had to match a picture of an animal to a sentence, the presence of a matching or mismatching object should not significantly influence one’s performance. However, if comprehenders routinely generate complete mental simulations during language comprehension, the presence of a mismatching object should create interference. If task instructions alone can influence what is included in a mental simulation, then this has significant consequences for the relevance of mental simulations in language comprehension, but if language comprehension requires mental simulations, then a complete simulation of all associated objects in a sentence should be performed automatically. If we are instructed, however, to only pay attention to one of the objects in the sentence, and subsequently, no interference is created when a mismatching object is shown, this would imply that a complete simulation is not required for comprehension. These are the questions that we attempt to answer with this study.

### This study

**Figure 1.**
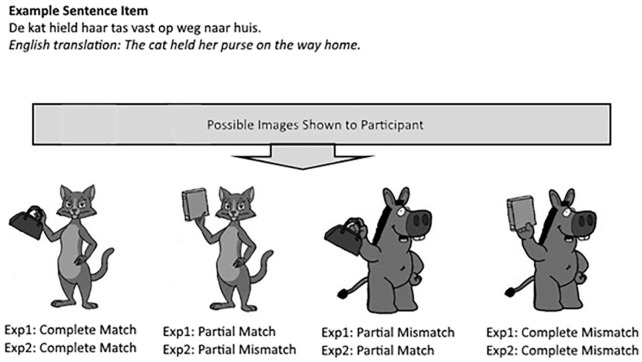
Example of an item in Experiments 1 and 2.

In Experiment 1, participants had to read sentences describing animals using a tool in some way. After they read a sentence, they saw a picture of a cartoon animal holding a tool, and they were instructed to answer whether the animal was mentioned in the previous sentence or not (see Figure 1 for an example). This picture either fully matched the preceding sentence (i.e., both the animal and the tool matched), partially matched (i.e., only the animal matched), partially mismatched (i.e., only the tool matched), or completely mismatched (i.e., neither tool nor animal matched). If participants only simulate the objects they are required to complete the task, then we would not expect any differences in response times between the complete and partial match conditions. However, based on the fact that participants can generate non-destructive interpretations of novel concepts and are able to do this fairly quickly (Connell & Lynott, 2011), we predict that participants do simulate both objects and, thus, will respond faster to pictures that completely match the sentence, compared to when they partially match. Furthermore, if the complete scene is simulated, it should be easier to reject a picture when there is absolutely no overlap between the picture and the mental simulation (i.e., a complete mismatch) compared to when there is some overlap (i.e., a partial mismatch). Under the assumption of complete simulation, partial overlap should create interference, and thus give rise to longer response times than no overlap. In summary, if participants respond fastest to the complete match condition compared to the partial match condition, this would be taken as evidence for object combinations in mental simulations and evidence for facilitation in the complete overlap condition. Moreover, if they respond fastest to the complete mismatch condition compared to the partial mismatch condition, this would be taken as evidence that partial overlap in a mental simulation generates interference.

In Experiment 2, participants received the exact same items as in Experiment 1, but were instructed instead to respond whether the tool in the picture was mentioned in the previous sentence or not. By having participants attend to different parts of the sentence, we can see whether instructions modulate the contents of mental simulations. It should be noted here that in the pictures, the tool occupies much less of the visual space than the animal in order for the picture to maintain a semblance of realism. As such, it is possible that facilitation effects in Experiment 2 are smaller than in Experiment 1 as the target is smaller. Similarly, however, it would also be possible to have more interference effects because the larger object (i.e., the animal) mismatches the sentence. Our predictions for Experiment 1 were also our predictions for Experiment 2, namely that response times in the complete match condition would be significantly shorter than in the partial match conditions. If Experiment 2 similarly illustrates that participants respond faster in the complete match condition compared to the partial match condition, then we will have found no evidence to suggest that task instruction moderates the effects.

### Ethics statement

The participation in all experiments was voluntary. The participants subscribed to the experiments online through the university platform, and were told that by signing up for a study, they declare to voluntarily participate in this study. They were briefed with the content of each study and provided written consent. Participants were told they were free to terminate the experiment at any point in time without experiencing negative consequences. This study was approved by the Ethics Committee of Psychology at the Erasmus University Rotterdam, The Netherlands.

### Preregistration

The predictions, exclusion criteria, design, methods, analyses, and materials of all the experiments reported in this article were pre-registered in advance of data collection and analysis on the Open Science Framework (OSF) to ensure confirmatory procedures were conducted according to a priori criteria, and can be viewed on https://osf.io/hqs7u. Analyses that were not pre-registered are referred to in this article under the heading “Exploratory Analyses.”

## Experiment 1

### Method

#### Participants

Based on a power-analysis of the effect size reported in the study by Zwaan et al. (2002), who found a significant match effect for shape, we found that at least 84 participants would be required to find an effect if it exists (*d* = 0.31, α = .05, Power = .80). To ensure our study was not underpowered after exclusions, we recruited 100 Dutch participants (*M_age_* = 20.58 years, *SD_age_* = 3.02 years, 87 females, 13 males) from the Bachelor of Psychology at the Erasmus University Rotterdam. Participants were excluded if their average accuracy was below 80%. As a result of this exclusion criterion data from 10 participants were excluded in the analyses, resulting in a sample of 90 participants.

#### Materials

For this study, we wanted to ensure that the situations described in the sentences would be unfamiliar to participants, as this would lead to the increased likelihood that the sentence would be simulated rather than just superficially processed. As such, we decided to include sentences that described animals performing actions on tools similar to what could be experienced in a cartoon story. Readers are known to adapt quickly to such a cartoon world (Nieuwland & Van Berkum, 2006). In total, 96 Dutch sentences were created that described an animal holding a certain object (e.g., *The dog walked with his umbrella across the street.*) and a total of 96 cartoon images were created for this study. There were 2 possible sentence versions (see Figure 1) and participants viewed only 1 of these versions (which were counterbalanced) and, therefore, saw in total, 48 sentence items and 48 images. Per sentence, participants also answered a comprehension question, to which they responded after seeing the image. The cartoon images were created using images of cartoon animals and of tools found on the Google search engine, which were subsequently edited using the Paint.NET software (version 4.1.5) to look as though the cartoon animal was holding the object. The images were displayed in greyscale (to ensure that effects of colour could not confound the results) and did not exceed a 300 × 300 pixel resolution (approximately 7.9 cm × 7.9 cm on-screen). The experiment was programmed using E-Prime 2.0 Professional and participants completed the experiments in isolated cubicles with computers equipped with 24.1″ TFT-IPS screens with a resolution of 1,920 × 1,200 and a ratio of 16:10.

#### Design and procedure

The study is a 2 (Type: partial vs. complete) × 2 (Match: match vs. mismatch) × 2 (Sentence: version 1 vs. version 2) × 4 (Image: version 1 vs. version 2 vs. version 3 vs. version 4) mixed design. “Type” and “Match” are within-subjects variables: participants viewed 12 images per type-match condition. Per sentence, there were four possible images that could be shown to the participant:

1. Complete match: correct animal + correct object → Participants give “yes” response;
2. Partial match: correct animal + wrong object → Participants give “yes” response;
3. Partial mismatch: wrong animal + correct object → Participants give “no” response;
4. Complete mismatch: wrong animal + wrong object → Participants give “no” response.

“Sentence” and “Image” are between-subjects variables which served to counterbalance this study, as for each sentence there were four possible images that could be shown to the participant, and there were two sentence versions. This led to a total of eight counterbalancing lists. An additional experiment from another study was performed by the participants in the same session, which was counterbalanced to be completed either before or after this study.

Participants were instructed that they would perform a self-paced reading task using the spacebar and that they would see a cartoon image after each sentence and were instructed to answer YES (the “L” key) if the cartoon animal matched the preceding sentence, and answer NO (the “A” key) if the cartoon animal mismatched the preceding sentence. After responding to the image, participants answered a comprehension question which asked what object the animal was holding, providing two answer options to the participants that they could respond to with the “L” and “A” keys. The purpose of the comprehension questions was to ensure that participants did not only read the name of the animal but that they properly read the entire sentence. Before starting the experiment, they received six practice items.

A trial looked as follows: participants saw the > symbol left-aligned in the centre of the screen for 1,000 ms. Subsequently, the sentence was shown left-aligned in the centre of the screen and remained on-screen until participants pressed the spacebar. Subsequently, a fixation cross appeared in the centre of the screen (centre-aligned) for 500 ms, after which the image appeared in the centre of the screen (centre-aligned) and remained on-screen until participants provided a response. After the response to the image they received the comprehension question: “What was the animal holding?” where two answer options were shown on opposite horizontal sides of the screen.

### Results

#### Data analysis

Participants with an average accuracy below 80% were excluded from the analyses. If items in the “complete match” condition had an accuracy score of less than 60%, that item (across all conditions) would have been excluded as well, but as the accuracy scores were above this threshold, zero items were excluded from the analyses. All response-time analyses were performed on correct responses only. Per participant, the median response time was taken per condition, as is common in sentence-picture verification studies (Hoeben Mannaert et al., 2017, 2019; Zwaan et al., 2002; Zwaan & Pecher, 2012) to prevent extreme values from influencing the data. To examine whether there is an interaction between the match and mismatch conditions, a repeated-measures analysis of variance (rmANOVA) was performed with Type (partial vs. complete) and Match (match vs. mismatch) included as the repeated-measures factors, and List was included as the between-subjects factor.

Important to note here is that the mismatch condition always required a “no” response, while the match condition always required a “yes” response. Even though the pictures were counterbalanced across conditions, in general “yes” responses tend to be faster than “no” responses (e.g., Brouillet et al., 2010). This has no significant consequences for testing our hypotheses, as the main result of interest is the difference between the complete and partial match condition, which both required a “yes” response.

Subject analyses are denoted with the subscript “1” and item analyses are denoted with the subscript “2.” Paired-samples *t*-tests were performed to examine the difference between the complete and partial match conditions and between the complete and partial mismatch conditions. The data can be viewed on https://osf.io/8z6xd.

#### Accuracy

The rmANOVA showed a significant effect of Type, illustrating that participants were significantly more accurate when the image completely matched or mismatched compared to when it partially matched or mismatched, *F*_1_(1,82) = 17.19, *p* < .001, $\eta_{p}^{2}=.17$; *F*_2_(1,47) = 14.26, *p* < .001, $\eta_{p}^{2}=.23$. There was also a significant match effect in the subject analyses (but not in the item analyses), meaning that, when the images showed the same animal as mentioned in the sentence, participants were significantly more accurate than when the animals differed, *F*_1_(1,82) = 4.41, *p* = .039, $\eta_{p}^{2}=.05$; *F*_2_(1,47) = 0.44, *p* = .513, $\eta_{p}^{2}=.01$. There was no significant interaction between Type and Match, *F*(1,82) = 1.42, *p* = .238, $\eta_{p}^{2}=.02$; *F*_2_(1,47) = 1.71, *p* = .197, $\eta_{p}^{2}=.04$. There was no significant between-subjects effect of List, *F*_1_(7,82) = 0.40, *p* = .898, $\eta_{p}^{2}=.03$, but there was a significant 3-way interaction with Type, List, and Match, *F*_1_(7,82) = 6.41, *p* < .001, $\eta_{p}^{2}=.35$.

A paired-samples *t*-test showed that participants were significantly more accurate in the complete match condition (*M* = 0.99, *SD* = 0.04) compared to the partial match condition, *M* = 0.97, *SD* = 0.06, *t*_1_(89) = 2.72, *p* = .008, Cohen’s *d* = 0.29, but this was not replicated in the item analysis, *t*_2_(47) = 1.93, *p* = .060. A second paired-samples *t*-test illustrated that participants were also significantly more accurate in the complete mismatch condition (*M* = 0.98, *SD* = 0.04) compared to the partial mismatch condition, *M* = 0.95, *SD* = 0.06, *t*(89) = 3.65, *p* < .001, Cohen’s *d* = 0.38; *t*_2_(47) = 3.21, *p* = .002.

#### Response times

Descriptive statistics can be seen in Table 1. The rmANOVA illustrated a significant effect of Type, showing that images that either completely matched or completely mismatched led to shorter response times compared to when they partially matched or mismatched, *F*_1_(1,82) = 10.22, *p* = .002, $\eta_{p}^{2}=.11$; *F*_2_(1,47) = 23.34, *p* < .001, $\eta_{p}^{2}=.33$. There was no significant effect of Match, *F*_1_(1,82) = 1.85, *p* = .178, $\eta_{p}^{2}=.02$; *F*_2_(1,47) = 0.16, *p* = .687, $\eta_{p}^{2}=.003$, nor a significant interaction between Type and Match, *F*_1_(1,82) = 0.01, *p* = .925, $\eta_{p}^{2}=.00$; *F*_2_(1,47) = 0.02, *p* = .878, $\eta_{p}^{2}=.001$. There was no significant between-subjects effect of List, *F*_1_(7,82) = 1.25, *p* = .288, $\eta_{p}^{2}=.10$, but there was a significant 3-way interaction with Type, List, and Match, *F*_1_(7,82) = 3.41, *p* = .003, $\eta_{p}^{2}=.23$.

**Table 1.** Descriptives Experiments 1 and 2.

Type	Match	*N*	Mean Accuracy (SD)	Mean Response Times (SD)
Experiment 1				
Partial	Match	90	0.97 (0.06)	1,461 (574)
	Mismatch	90	0.95 (0.06)	1,422 (549)
Complete	Match	90	0.99 (0.04)	1,387 (557)
	Mismatch	90	0.98 (0.04)	1,358 (561)
Experiment 2				
Partial	Match	92	0.92 (0.16)	1,687 (623)
	Mismatch	92	0.98 (0.07)	1,622 (627)
Complete	Match	92	0.98 (0.05)	1,501 (545)
	Mismatch	92	0.99 (0.04)	1,560 (568)

SD: standard deviation.

Paired-samples *t*-tests were performed to see whether there was a significant difference between the complete match and partial match conditions (which both required *yes* responses), and between the complete mismatch and partial mismatch conditions (which both required *no* responses). The analyses showed that participants responded significantly faster when there was a complete match (*M* = 1,387 ms, *SD* = 557 ms) compared to when there was a partial match, *M* = 1,461 ms, *SD* = 574 ms, *t*_1_(89) = −2.08, *p* = .040, Cohen’s *d* = −0.22; *t*_2_(47) = −3.17, *p* = .003. Moreover, participants also responded significantly faster when there was a complete mismatch (*M* = 1,358 ms, *SD* = 561 ms) compared to when there was a partial mismatch, *M* = 1,422, *SD* = 549, *t*_1_(89) = −2.59, *p* = .011, Cohen’s *d* = −0.27; *t*_2_(47) = −2.63, *p* = .011.

#### Exploratory analyses

To examine the three-way interaction between Type, List, and Match, a simple main effects analysis was done with “Type” as the Simple Effects Factor, and List and Match as moderator factors for both response times and accuracy scores. The simple effects analysis for response times illustrated a significant difference between the partial and complete mismatch condition for List G, *F*(1) = 7.61, *p* = .020, in the opposite direction as the other lists. So the participants in this List responded significantly faster in the partial mismatch condition compared to the complete mismatch condition. For the accuracy scores, however, no such effect was found for List G. In fact, for the accuracy scores, a reverse pattern of effects was found only for List D, though this did not reach significance in the analysis, *F*(1) = 4.81, *p* = .053. It is difficult to ascertain what caused this interaction, given that the same animals and tools were used in each list, albeit using different combinations. If the interaction effect was simply due to having several bad items in one condition, then spurious effects should be found across multiple lists. As the main focus of this study is on the effects in the match condition, this interaction effect does not affect our main conclusions.

#### Comprehension accuracy

Participants on average had high comprehension accuracy (*M* = 0.87, *SD* = 0.20), suggesting that they did properly read the sentences in their entirety. An rmANOVA was performed on the comprehension accuracy data and found no significant main effect of Type, *F*(1,82) = 0.41, *p* = .526, nor a significant main effect of Match, *F*(1,82) = 0.30, *p* = .583, but found a significant interaction between Type and Match, *F*(1,82) = 34.71, *p* < .001. There was no between-subjects effect of List, *F*(7,82) = 0.63, *p* = .733. Paired-samples *t-*tests with a Bonferroni-adjusted alpha (α = .025) show that participants responded more accurately to the question “What was the animal holding?” in the partial mismatch condition (*M* = 0.99, *SD* = 0.03) compared to the complete mismatch condition, *M* = 0.75, *SD* = 0.40, *t*(89) = −5.89, *p* < .001, and responded more accurately in the complete match condition (*M* = 0.99, *SD* = 0.03) compared to the partial match condition, *M* = 0.74, *SD* = 0.40, *t*(89) = 5.95, *p* < .001.

### Discussion

The aim of Experiment 1 was to see whether participants combined multiple objects in their mental simulations. The results showed that participants were significantly faster when there was a complete match compared to a partial match, suggesting that comprehenders simulate multiple objects during language comprehension. The findings further suggest that during this sentence-picture verification task, participants compare what is currently being simulated with what is shown in the picture. When there was a complete mismatch (i.e., no overlap) with the preceding sentence, participants were significantly more accurate and faster compared to when there was only a partial mismatch (i.e., only the object held by the animal matched). This provides support for the idea that when there is complete overlap between simulation and image, there is facilitation of the participant’s response. Similarly, it is equally easy to identify when there is completely zero overlap between simulation and image. However, as soon as one of the objects in the picture overlaps with the simulation while the other does not, there appears to be interference, which can be seen in both the decreased accuracy scores and the increased response times. The interpretation of the accuracy scores, however, should be taken with caution, as the accuracy scores overall were nearly 100% and the percentage differences between conditions were between only 2% and 3%. Such small differences in accuracy may, therefore, not be very meaningful.

When examining the accuracy responses to the comprehension question “what was the animal holding?” participants on average had high accuracy scores, suggesting that they properly read the sentences. Interestingly, the participants performed best in the partial mismatch and complete match conditions, meaning that when the preceding image showed the correct tool it facilitated their response, and if it showed the incorrect tool it interfered with their response. It is possible that once they were presented with the comprehension question, they could no longer remember whether it was the sentence or the image that contained the correct answer to the comprehension question.

## Experiment 2

Experiment 1 showed that participants respond significantly faster when the picture completely matches what was stated by the sentence compared to when it only partially matched. This suggests that we indeed combine objects in mental simulations during language comprehension. Experiment 2 was conducted to examine whether participants still show the same match effect as in Experiment 1 when participants are instructed to respond to the *tool* as opposed to the *animal*. Experiment 2, therefore, is a conceptual replication of Experiment 1. If the results from Experiment 2 do not replicate those of Experiment 1, this would suggest that task instructions can modulate the contents of mental simulations. Specifically, if asking participants to respond only to the *tool* leads to only the tool being mentally simulated, this would lead to no significant differences in response times between the complete match and partial match conditions. However, if changing the instructions does not lead to different results compared to Experiment 1, this would mean that comprehenders routinely generate complete mental simulations of texts.

### Method

#### Participants

We recruited 100 Dutch participants (*M_age_* = 20.42 years, *SD_age_* = 3.72 years, 80 females, 20 males) from the Bachelor of Psychology at the Erasmus University Rotterdam. Participants were excluded if their average accuracy was below 80%, as a result of these exclusion criteria, data from 8 participants were excluded in the analyses, resulting in a sample of 92 participants.

#### Materials

The same materials were used as in Experiment 1.

#### Design and procedure

The design of Experiment 2 was identical to that of Experiment 1. The only difference in the procedure was that participants were instructed to respond “YES” (the “L” key) if the *tool* matched the one described in the sentence, and to respond “NO” (the “A” key) if it mismatched. As a result, the following four image types could be shown to the participants:

5. Complete match: correct object + correct animal → Participants give “yes” response;
6. Partial match: correct object + wrong animal → Participants give “yes” response;
7. Partial mismatch: wrong object + correct animal → Participants give “no” response;
8. Complete mismatch: wrong object + wrong animal → Participants give “no” response.

### Results

#### Data analysis

The same analyses that were performed on the data of Experiment 1 were performed on the data from Experiment 2.

#### Accuracy

The rmANOVA showed a significant main effect of Type, *F*_1_(1,91) = 20.78, *p* < .001, $\eta_{p}^{2}=.19$; *F*_2_(1,47) = 49.32, *p* < .001, $\eta_{p}^{2}=.51$, a main Match effect, *F*_1_(1,91) = 10.53, *p* = .002, $\eta_{p}^{2}=.10$; *F*_2_(1,47) = 53.84, *p* < .001, $\eta_{p}^{2}=.53$, and a significant interaction between Type and Match, *F*_1_(1,91) = 8.13, *p* = .005, $\eta_{p}^{2}=.08$; *F*_2_(1,47) = 30.46, *p* < .001, $\eta_{p}^{2}=.39$. List did not interact with any of the variables and, therefore, was excluded from the rmANOVA. A paired-samples *t*-test showed that participants were significantly more accurate in the complete match condition (*M* = 0.98, *SD* = 0.05) compared to the partial match condition, *M* = 0.92, *SD* = 0.16, *t*_1_(91) = 3.93, *p* < .001, Cohen’s *d* = 0.41; *t*_2_(47) = 7.36, *p* < .001. A second paired-samples *t*-test illustrated that participants were also significantly more accurate in the complete mismatch condition (*M* = 0.99, *SD* = 0.04) compared to the partial mismatch condition, *M* = 0.98, *SD* = 0.07, *t*_1_(91) = 2.61, *p* = .011, Cohen’s *d* = 0.27; *t*_2_(47) = 2.79, *p* = .008.

#### Response times

The rmANOVA illustrated a significant effect of Type, showing that images that either com-pletely matched or completely mismatched led to shorter response times compared to when they partially matched or mismatched, *F*_1_(1,84) = 28.61, *p* < .001, $\eta_{p}^{2}=.25$; *F*_2_(1,47) = 14.09, *p* < .001, $\eta_{p}^{2}=.23$. There was no significant effect of Match, *F*_1_(1,84) = 0.05, *p* = .821, $\eta_{p}^{2}=.001$; *F*_2_(1,47) = 0.10, *p* = .750, $\eta_{p}^{2}=.002$, but there was a significant interaction between Type and Match, *F*_1_(1,84) = 4.91, *p* = .029, $\eta_{p}^{2}=.06$; *F*_2_(1,47) = 6.24, *p* = .016, $\eta_{p}^{2}=.12$. There was no significant between-subjects effect of List, *F*_1_(7,84) = 0.76, *p* = .620, $\eta_{p}^{2}=.06$, but there was a significant interaction between List and Match, *F*_1_(7,84) = 2.14, *p* = .048, $\eta_{p}^{2}=.15$.

Paired-samples *t*-tests were performed to see whether there was a significant difference between the complete match and partial match conditions, and between the complete mismatch and partial mismatch conditions. The analyses showed that participants responded significantly faster when there was a complete match (*M* = 1,501 ms, *SD* = 545 ms) compared to when there was a partial match, *M* = 1,687 ms, *SD* = 623 ms, *t*_1_(91) = −4.82, *p* < .001, Cohen’s *d* = −0.50; *t*_2_(47) = −3.96, *p* < .001. Interestingly, participants did not respond significantly faster when there was a complete mismatch (*M* = 1,560 ms, *SD* = 568 ms) compared to when there was a partial mismatch, *M* = 1,622 ms, *SD* = 627 ms, *t*_1_(91) = −1.86, *p* = .067, Cohen’s *d* = −0.19; *t*_2_(47) = −0.95, *p* = .347.

#### Comprehension accuracy

Participants on average had high comprehension accuracy (*M* = 0.82, *SD* = 0.21), suggesting that they did properly read the sentences in their entirety. An rmANOVA was performed on the comprehension accuracy data and found a significant main effect of Type, *F*_1_(1,84) = 7.69, *p* = .007, a significant main effect of Match, *F*_1_(1,84) = 6.99, *p* = .010, and a significant interaction between Type and Match, *F*_1_(1,84) = 52.47, *p* < .001. There was no between-subjects effect of List, *F*_1_(7,84) = 0.58, *p* = .769. Paired-samples *t-*tests with a Bonferroni-corrected alpha (α = .025) show that participants responded more accurately to the question “which animal was holding the tool?” in the partial mismatch condition (*M* = 0.97, *SD* = 0.09) compared to the complete mismatch condition, *M* = 0.69, *SD* = 0.41, *t*(91) = −6.52, *p* < .001, and responded more accurately in the complete match condition (*M* = 0.98, *SD* = 0.06) compared to the partial match condition, *M* = 0.64, *SD* = 0.42, *t*(91) = 7.89, *p* < .001.

#### Exploratory analyses

The analyses in this section were not pre-registered online before data collection had started. A random-effects meta-analysis was performed using the *metafor* (Viechtbauer, 2010) package in *R* (version 3.6.0) to compare the facilitation effect (i.e., the difference between the complete and partial match conditions) and the interference effect (i.e., the difference between the complete and partial mismatch conditions) across Experiments 1 and 2. The code used in *R* for the analyses can be found in the online Supplementary Material. The meta-analysis for the facilitation effect showed that participants across Experiments 1 and 2 responded 129 ms (95% CI = [19 ms, 239 ms]) faster when there was a complete match compared to a partial match (*p* = .021). Heterogeneity was significant, *Q*(1) = 4.56, *p* = .033, indicating that the facilitation effect was larger in Experiment 2 than in Experiment 1. The meta-analysis for the interference effect illustrated that participants across Experiments 1 and 2 responded 63 ms (95% CI = [24 ms, 102 ms]) faster when there was a complete mismatch compared to when there was a partial mismatch (*p* = .002). Heterogeneity was not significant, *Q*(1) = 0.002, *p* = .962.

### Discussion

The aim of Experiment 2 was to replicate the findings of Experiment 1 using the same method except for providing different instructions. In the current experiment, participants were told to respond to the object that was in the cartoon animal’s hand. The results of the analyses showed that participants were significantly faster when the picture completely matched what was stated in the sentence compared to when it partially matched. There was no significant difference between the complete mismatch and partial mismatch conditions. The effect in response times found in the match condition and the lack of effect in the mismatch condition is what drove the interaction in the rmANOVA. A simple main effects analysis confirmed that, indeed, participants responded significantly faster in the complete match condition compared to the partial match condition, while no significant differences are present within the mismatch condition.

Participants were also significantly more accurate when the image was either completely matching or completely mismatching the preceding sentence compared to when they were only partially matching or mismatching. As with Experiment 1, however, these small percentage differences are limited in their meaningfulness and should be interpreted with caution.

The meta-analysis supports the hypothesis that multiple objects are mentally simulated during language comprehension as when both objects were shown in the image there was clear facilitation of the response, as seen by the 129 ms facilitation effect across both experiments. Moreover, when 1 of the 2 components of the image matched the sentence when the target item mismatched, there was a 63 ms interference effect across both experiments.

Comprehension accuracy was highest in the partial mismatch condition compared to the complete mismatch condition, and in the complete match condition compared to the partial match condition. These findings illustrate that participants responded more accurately when the preceding image portrayed the correct animal. It is possible that once they were presented with the comprehension question, they could no longer remember whether it was the sentence or the image that contained the correct answer to the comprehension question.

## General discussion

Much research until now has focused on which object properties are present in mental simulations, but none have examined whether multiple objects can be combined in a mental simulation. This study aimed to discover whether comprehenders combine multiple objects in their mental simulations, and whether this is dependent on task instructions. In Experiment 1, participants responded to images of cartoon animals holding a tool and were asked to response affirmatively if the pictured cartoon animal matched the animal described by the previous sentence. In Experiment 2, participants responded to the same stimuli but instead had to respond affirmatively if the pictured tool matched the tool described by the previous sentence. The images shown could either completely match, partially match, completely mismatch, or partially mismatch the preceding sentence.

The findings from both Experiments 1 and 2 provide support for our prediction that participants would respond faster when there was a complete match compared to a partial match. These findings are in line with a study by Šetić and Domijan (2017), who found that when sentences describe a multiple of the same object (e.g., two dogs), this is also represented in mental simulations. Together, these studies can be taken as evidence that comprehenders combine multiple objects in their mental simulations during language comprehension. If they would not combine objects in a mental simulation, there would be no difference between the complete match and partial match conditions, as participants were only instructed to respond to one of the objects in the image (either the cartoon animal or the tool), and, thus, would not have needed to simulate the other object for the sake of the task. Therefore, task instructions also do not seem to modulate the contents of mental simulations.

These findings run counter to what was found by Lebois et al. (2015), who observed that task instructions can influence concept activation. However, it is similarly possible that the nature of our task caused both animal and tool to be simulated, as participants also had to respond to comprehension questions about the sentences, thus perhaps directing them to simulate both objects. We believe, however, that the comprehension questions only ensured that participants actually read each sentence, rather than stopping once they had read the name of the object which had to be verified in the subsequent picture. Although it may be of interest for future studies to explore the influence of comprehension questions on mental simulations, this was outside of the scope of this study.

An additional finding from this study was that in Experiment 1, participants responded significantly faster when there was a complete mismatch compared to a partial mismatch, but this was not replicated in Experiment 2, although the difference was in the predicted direction. A meta-analysis performed on the data from both experiments nonetheless found a significant interference effect, suggesting that when one of the objects matches what is mentally simulated, while the target mismatches, interference is generated. We hypothesised that comprehenders compare the contents of their mental simulation to the image during a sentence-picture verification task. Thus, when there is complete overlap between the pictured image and the mental simulation, facilitation occurs. Similarly, when there is no overlap whatsoever, it is very easy to respond that the image does not represent the previous sentence. However, if there is only a partial overlap, interference occurs. It is likely that the reason Experiment 2 did not replicate this finding is because in Experiment 2 participants were asked to react to the tool in the picture, which was smaller in size compared to the cartoon animal. If it were simply due to the fact that the agent in the text is more salient than the instrument, we should have seen no significant difference in the match condition either. As it is, it seems more likely that the interaction here is driven by the differences in object sizes in the picture. Perhaps, if our experiment had used larger images, this effect would also have been replicated, but this is something that could be investigated in future replications.

The ECCo theory proposed by Lynott and Connell (2010) proposes that during conceptual combinations, compound nouns can either be simulated in a destructive (where one of the concepts is reduced) or a non-destructive manner (where both concepts remain intact in the simulation) and that the linguistic system interacts with the simulation system to determine how these concepts need to be combined. The study by Connell and Lynott (2011: 4) showed that it is easier for comprehenders to create non-destructive interpretations (i.e., for “octopus apartment,” the interpretation “a place where an octopus might live” is considered non-destructive) than it is to create destructive interpretations (i.e., “an apartment with eight rooms” requires the reduction of one of the properties in the compound noun). The finding in our study that objects can be combined in mental simulations fall in line with what is proposed to happen in a non-destructive conceptual combination. As conceptual combinations have not yet been tested using a sentence-picture verification paradigm, our study is the first to suggest that this link between visual representations and conceptual combinations actually exists in mental simulations.

Furthermore, in the “Introduction” section we argued that, if participants read a sentence containing two objects (i.e., an animal and a tool), but only simulate one of them (i.e., the object requiring a response), it could be argued that a complete mental simulation is not required for language comprehension. If language comprehension occurs even without a complete mental simulation, this would imply that language comprehension does not require mental simulations. By showing that comprehenders do represent both objects in their mental simulation, regardless of task instructions, we provide preliminary evidence that a complete mental simulation becomes activated (and perhaps even is required) during language comprehension.

An alternative explanation for the findings in this study is given by Proctor and Cho’s (2006) polarity correspondence principle, which argues that poles of bipolar dimensions are coded as a “+” or “–.” This means that when stimulus and response match that response times are facilitated. Specifically, if “yes” responses and “matches” are mapped onto the “+” pole and “no” responses and “mismatches” to the “–” pole, then facilitation occurs when there is a complete match (+++) compared to when there is only a partial match (++−). Similarly, when there is a complete mismatch (−−−) responses are similarly facilitated compared to when there is a partial mismatch (−−+). This explanation and theories of grounded cognition are not, however, mutually exclusive. To place the match and mismatch conditions on these separate poles the picture has to be categorised as a match or a mismatch first, and the only way this can be done is by engaging the simulation system first. As such, although the polarity correspondence principle can partially explain the mechanism behind the match effect, it cannot account for the sensorimotor activation required for mental simulations.

A potential limitation to the two experiments is the fact that we did not counterbalance which hand was responsible for which type of response. In this study, all “yes” responses were performed using the right hand and all “no” responses were performed using the left hand. Using such a counterbalancing measure in this study would have meant using 16 counterbalancing lists, which was not feasible. Future studies interested in further examining the differences between the partial match and partial mismatch condition would have to also counterbalance hand use.

To conclude, studies on mental simulations have thus far only measured the activation of only a single object property in mental simulations during language comprehension. This study examined whether multiple objects are combined in mental simulations and found support that this is indeed the case, regardless of task instructions. These results are in line with studies that have examined conceptual combinations in mental simulations. Future research should continue to focus on how mental simulations are constructed in novel and familiar contexts, and how they may be further altered by new incoming information.

## Supplemental Material

QJE-STD-19-167.R1-Supplementary_Material – Supplemental material for Object combination in mental simulations Click here for additional data file. Supplemental material, QJE-STD-19-167.R1-Supplementary_Material for Object combination in mental simulations by Lara N Hoeben Mannaert, Katinka Dijkstra and Rolf A Zwaan in Quarterly Journal of Experimental Psychology
