# Supplementary material for: Object combination in mental simulations
Source: Q J Exp Psychol (Hove). 2020 Jun 18;73(11):1796–806. doi: 10.1177/1747021820933214 (PMC7583445; doi:10.1177/1747021820933214)
Supplement: QJE-STD-19-167.R1-Supplementary_Material – Supplemental material for Object combination in mental simulations [file QJE-STD-19-167.R1-Supplementary_Material.docx]

Supplementary Material for:

Object combination in mental simulations

Lara N. Hoeben Mannaert, Katinka Dijkstra, and Rolf A. Zwaan

library(metafor)

# Definition of vectors

version <- c("match", "match", "mismatch", "mismatch")

experiment <- c("Experiment 1", "Experiment 2", "Experiment 1", "Experiment 2")

mpartial <- c(1461, 1687, 1422, 1622)

sdpartial <- c(574, 623, 549, 627)

mcomplete <- c(1387, 1501, 1358, 1560)

sdcomplete <- c(557, 545, 561, 568)

n <- c(90, 92, 90, 92)

corr <- c(.82, .81, .91, .86)

#Creation of data frame

data <- data.frame(version, experiment, mpartial, sdpartial, mcomplete, sdcomplete, n, corr, stringsAsFactors = FALSE)

data

#Random-effects meta-analysis of Match condition

dat <- escalc(measure="MC", m1i=mpartial, sd1i=sdpartial, m2i=mcomplete, sd2i=sdcomplete, ni=n, ri=corr,

data=data, subset=(version=="match"))

res.match <- rma(yi, vi, data=dat)

res.match

#Random-effects meta-analysis of Mismatch condition

dat <- escalc(measure="MC", m1i=mpartial, sd1i=sdpartial, m2i=mcomplete, sd2i=sdcomplete, ni=n, ri=corr,

data=data, subset=(version=="mismatch"))

res.mismatch <- rma(yi, vi, data=dat)

res.mismatch
